# Factors Associated with Unfavorable Treatment Outcomes in New and Previously Treated TB Patients in Uzbekistan: A Five Year Countrywide Study

**DOI:** 10.1371/journal.pone.0128907

**Published:** 2015-06-15

**Authors:** Jamshid Gadoev, Damin Asadov, Mirzagolib Tillashaykhov, Katie Tayler-Smith, Petros Isaakidis, Andrei Dadu, Pierpaolo de Colombani, Sven Gudmund Hinderaker, Nargiza Parpieva, Dilrabo Ulmasova, Avazbek Jalolov, Atadjan Hamraev, Engy Ali, Martin van den Boom, Asmus Hammerich, Ogtay Gozalov, Masoud Dara

**Affiliations:** 1 World Health Organization country office in Tashkent, Tashkent, Uzbekistan; 2 State Post Graduate Institute of Medical Education, Tashkent, Uzbekistan; 3 Republican Specialized TB center, Tashkent, Uzbekistan; 4 Medecins Sans Frontieres (MSF), Operational Center Brussels, Operational Research Unit, MSF-Luxembourg, Luxembourg, Luxembourg; 5 WHO Regional Office for Europe, TBM department, Copenhagen, Denmark; 6 Centre for International Health, University of Bergen, Bergen, Norway; 7 Tashkent Medical Academy, Tashkent, Uzbekistan; 8 Republican DOTS center, Tashkent, Uzbekistan; 9 Nukus branch of Tashkent State Pediatric Institute, Nukus, Karakalpakstan; Universidad Nacional de La Plata., ARGENTINA

## Abstract

**Background:**

TB is one of the main health priorities in Uzbekistan and relatively high rates of unfavorable treatment outcomes have recently been reported. This requires closer analysis to explain the reasons and recommend interventions to improve the situation. Thus, by using countrywide data this study sought to determine trends in unfavorable outcomes (lost-to-follow-ups, deaths and treatment failures) and describe their associations with socio-demographic and clinical factors.

**Method:**

A countrywide retrospective cohort study of all new and previously treated TB patients registered in the National Tuberculosis programme between January 2006 and December 2010.

**Results:**

Among 107,380 registered patients, 67% were adults, with smaller proportions of children (10%), adolescents (4%) and elderly patients (19%). Sixty per cent were male, 66% lived in rural areas, 1% were HIV-infected and 1% had a history of imprisonment. Pulmonary TB (PTB) was present in 77%, of which 43% were smear-positive and 53% were smear-negative. Overall, 83% of patients were successfully treated, 6% died, 6% were lost-to-follow-up, 3% failed treatment and 2% transferred out. Factors associated with death included being above 55 years of age, HIV-positive, sputum smear positive, previously treated, jobless and living in certain provinces. Factors associated with lost-to-follow-up were being male, previously treated, jobless, living in an urban area, and living in certain provinces. Having smear-positive PTB, being an adolescent, being urban population, being HIV-negative, previously treated, jobless and residing in particular provinces were associated with treatment failure.

**Conclusion:**

Overall, 83% treatment success rate was achieved. However, our study findings highlight the need to improve TB services for certain vulnerable groups and in specific areas of the country. They also emphasize the need to develop unified monitoring and evaluation tools for drug-susceptible and drug-resistant TB, and call for better TB surveillance and coordination between provinces and neighbouring countries.

## Introduction

Tuberculosis (TB) remains a public health challenge worldwide and particularly in Central Asian countries. TB is one of the main health priorities in Uzbekistan and since 2004 the DOTS (directly observed treatment, short course) strategy has been progressively rolled out in the country. A recent study conducted in Tashkent, the capital city of Uzbekistan, showed that, of 1087 pulmonary TB patients started on treatment in 2005, 228 (21%) were lost to follow up [1]. Treatment failure among TB patients in certain provinces in Uzbekistan has also been relatively high (over 5–8%), and the prevalence of multidrug resistant TB (MDR TB) among new cases has tended to increase over the years (Drug resistance survey—14.2% in 2005 and 23.2% in 2011), [2, 3, 4].

Given the high rate of unfavorable treatment outcomes reported in some provinces, there is a need to analyze unfavorable outcomes countrywide and identify possible trends and associated risk factors that could guide the National TB programme (NTP) in further improvements. The NTP uses an individual-patient electronic database and this therefore allows for detailed analysis beyond the conventional monitoring and evaluation reports that are reliant on aggregate data. All TB patients registered in electronic database were on first-line treatment regimen. Of the drugs, isoniazid (H), rifampin (R), ethambutol (E), and pyrazinamide (Z) are considered first-line anti-TB drugs and form the core of standard treatment regimens for drug susceptible TB patients

Using countrywide TB data from Uzbekistan on TB patients receiving first-line treatment, the aim of our research was to determine a) trends in lost to follow-up, deaths and treatment failures between 2006 and 2010 and b) the socio-demographic and clinical risk factors associated with each of these unfavorable treatment outcomes.

## Methods

### Ethics

The study was approved by the “National Ethics Committee and Review Board” under the Ministry of Health (MoH) of Republic of Uzbekistan. The study satisfied the criteria for reports using routinely collected programmatic data set by the Médecins Sans Frontières Ethics Review Board (ERB), Geneva, Switzerland. Patient identifying information was removed prior to analysis. As this was a study of routinely collected monitoring data, patient consent was not required.

### Study design

This was a retrospective cohort study of routinely collected NTP data for all TB patients registered and commenced on first-line treatment between January 2006 and December 2010. All patients were followed up until the end of treatment (6–8 months) to ascertain treatment outcomes.

### Study setting

Previously part of the former Soviet Union, Uzbekistan is a country in Central Asia with an estimated population of more than 30 million. The country is divided into twelve provinces, the Republic of Karakalpakstan and the metropolitan area of Tashkent, the capital city.

#### The National TB Program (NTP)

TB control activities are coordinated countrywide by the Republican Specialized Scientific Practical Medical Center of Phthisiology and Pulmonology (RSSPMCPP), which is essentially the NTP. TB diagnosis and treatment are provided free of charge within the NTP–there are no private TB services. Nonetheless, first and second line anti-TB drugs are available on the open market as a result of there being no governmental regulations to forbid the selling of these drugs. All registered TB patients receive treatment in accordance with the Stop TB Strategy. The latest WHO Global TB Report [5] reports that only 35–50% of the national cases in Uzbekistan were detected between 2005 and 2010, implying that there may be a large number of TB cases left untreated or receiving ‘unregistered’ inefficacious treatments regimens in the country.

#### TB diagnosis and treatment

An established TB laboratory network in the country includes two National Reference Laboratories (NRL), five bacteriological laboratories and more than 300 smear microscopy laboratories, the latter of which perform direct microscopy of sputum collected in primary healthcare facilities. The mainstay of TB diagnosis in most provinces in Uzbekistan was through sputum smear microscopy or X-ray investigations. In accord with national guidelines [6], TB type was categorized as either pulmonary TB (PTB) or extrapulmonary TB (EPTB). PTB was defined as TB lesions involving the lung parenchyma, while TB lesions of the intra-thoracic lymph nodes (mediastinal and /or hilar), or tuberculosis pleurisies in the absence of radiographic changes in the lungs, were considered to be EPTB. If a patient presented with PTB and EPTB, they were recorded as having PTB when the pulmonary TB lesions were prominent; if however the patient had severe EPTB lesions (e.g. tuberculous meningitis) with limited forms of PTB lesions (smear-negative PTB), the patient was recorded as having EPTB.

Drug susceptibility testing (DST) during the study period could only be performed in two laboratories in Tashkent and Nukus. DST was performed using solid and liquid culture media and line probe assay (LPA) tests to determine type of drug resistance. Quality control in the two laboratories was ensured through routine checks by the Supra National Reference Laboratories (SNRL) in Borstel and Gauting, Germany. During the study period MDR-TB treatment was only available in Tashkent city, Nukus and the penitentiary system, and on account of limited bed capacity and resources, access to such treatment was not available for people residing in other provinces.

At the provincial level, TB treatment is provided under the supervision of the MoH and NTP at TB hospitals; at the district level, TB treatment is overseen by the TB dispensaries (outpatient care during the intensive phase and/or continuation phase of treatment) and also at the primary health care level (continuation phase of treatment) for both drug susceptible and MDR-TB patients in pilot areas. The Global Fund to Fight AIDS, Tuberculosis and Malaria (The Global Fund) provides all first-line anti-TB drugs and, since 2013, has provided all second-line drugs countrywide for treatment of drug resistant tuberculosis (DR TB). A recent Drug Resistance Survey (DRS) showed high rates of MDR TB among new and previously treated patients, 23% and 62% respectively (3). In response to the high levels of MDR TB, the NTP developed the “Updated National Plan on prevention and control of M/XDR TB for 2012–2015 in Uzbekistan” [7] which is in line with the “The Consolidated Action Plan to Prevent and Combat Multidrug- and Extensively Drug-Resistant Tuberculosis in the WHO European Region, 2011–2015”. This plan aims to decrease by 20 percentage points the proportion of MDR-TB among previously treated patients by the end of 2015; to diagnose at least 85% of all estimated MDR-TB patients by the end of 2015; to treat successfully at least 75% of all patients notified as having MDR-TB by the end of 2015. [8]

#### NTP monitoring system

Since 2005, an Epi-Info based-TB-ESCM (Tuberculosis Electronic Surveillance and Case Management) system has been implemented countrywide for disease surveillance and case management. All diagnosed patients are individually recorded in this electronic register with a unique registration number, and all of their clinical data are captured here.

TB treatment outcomes are in accord with WHO recommendations and described in national guidelines [6].

### Study population

The study population included all TB patients on first line treatment (new and retreatment cases) registered countrywide in the NTP between January 2006 and December 2010.

### Data collection and analysis

All data pertaining to this study were sourced from the TB ESCM electronic register.

Patient characteristics were reported using summary statistics. Age categories were defined as follows: less than 15 years–child; 15–18 years–adolescent, 19–55 years–adult; 56 years or above–elderly. Unfavorable treatment outcomes were defined as death, treatment failure and lost to follow up, and each of these outcomes was assessed separately. Risk factors for unfavorable TB treatment outcomes were determined by crude odds ratios (ORs) and adjusted ORs, comparing the odds of having that outcome of interest with the odds of not having that outcome of interest (i.e. having any other outcome). Adjusted ORs were determined through multivariate logistic regression using a backward stepwise elimination approach until all remaining variables in the model were significant at *P* = 0.05 or less. All related *P*-values were based on the Walds test and 95% confidence intervals were used throughout. Due to the incomplete ascertainment of transfer outs (many of which were considered to be failures secondary to MDR-TB), a sensitivity analysis was run in which all transfer outs were assumed to be failures.

The study was carried out between June 2013 and June 2014 using EpiData Analysis software (version 2.2.2.182, EpiData Association, Odense, Denmark) and STATA/IC 11 software (Stata corporation, College Station, Texas 77845, USA).

## Results

### Characteristics of the study population

Between 2006 and 2010, 110,146 TB patients on first-line drug regimen were registered in Uzbekistan. Of these, one percent (1226) had treatment outcome missing and 1.4% (1540) did not have a confirmed TB diagnosis; as such these patients were excluded from the analysis. Table 1 shows the baseline demographic and clinical characteristics of the 107,380 patients included in the study. Adults (19–55 years) made up 67% (71522) of the patients, while 10% (11519) were children (<15 years) and 4% (4764) were adolescents (15–18 years). Almost 60% were male and 66% from rural areas (70705). New patients made up 75% (81016) of the caseload, while 25% (26364) were previously treated patients. One percent (984) of patients was human immunodeficiency virus (HIV) positive and less than one percent (586) had a history of imprisonment. Seventy seven percent (82686) of patients had pulmonary tuberculosis (PTB), of which 43% (35178) were sputum smear positive, 53% (44205) sputum smear negative and 3303 with no or unknown sputum results.

**Table 1:** Socio-demographic and clinical characteristics and treatment outcomes of tuberculosis patients, Uzbekistan 2006–2010.

*Variables*	n (%)
**Total**	**107380**
**Age (years)**	
Children (<15)	11519 (11)
Adolescent (15–18)	4764 (4)
Adults (19–55)	71522 (67)
Elderly patients (>55)	19575 (18)
**Sex**	
Male	63724 (59)
Female	43656 (41)
**Place of residence**	
Urban	32752 (30)
Rural	70705 (66)
Unknown	3923 (4)
**Provinces**	
Republic of Karakalpakstan	13905 (13)
Tashkent city	8504 (8)
Andijan province	8525 (8)
Bukhara province	4857 (4)
Jizzakh province	4873 (5)
Kashkadarya province	8593 (8)
Navoi province	4258 (4)
Namangan province	8062 (8)
Samarkand province	11202 (10)
Surkhandarya province	5455 (5)
Syrdarya province	2601 (3)
Tashkent province	11314 (11)
Fergana province	9830 (9)
Khorezm province	5164 (5)
Navoi mining company	229 (<1)
Unknown	8 (<1)
**HIV status**	
HIV positive	984 (1)
HIV negative	96524 (90)
Unknown	9872 (9)
**TB type**	
PTB	
Smear positive	35178 (33)
Smear negative	44205 (41)
No sputum/no sputum result	3303 (3)
EPTB	24694 (23)
**History of TB treatment**	
New cases	81016 (75)
Retreatment cases	26364 (25)
**Treatment category**	
0	93 (<1)
I	76548 (71)
II	25986 (24)
III	4752 (4)
Unknown	1 (<1)
**History of contact with TB patient**	
No	90673 (85)
Yes	5590 (5)
Unknown	11117 (10)
**History of imprisonment**	
No	44147 (41)
Yes	586 (<1)
Unknown	62647 (58)
**Occupational status**	
Worker	12105 (11)
Jobless	48569 (45)
Pre-school age	2994 (3)
Pupil/student	11186 (10)
Pensioner	13898 (13)
Handicapped	5996 (6)
Unknown	12632 (12)
**Treatment outcomes**	
Cured	25404 (23)
Treatment completed	64218 (60)
Died	5953 (6)
Loss to follow up	6768 (6)
Failure	3312 (3)
Transferred out	1725 (2)

TB, Tuberculosis; PTB, Pulmonary TB; EPTB, Extrapulmonary TB.

### Treatment outcomes and their trends

**Fig 1:**
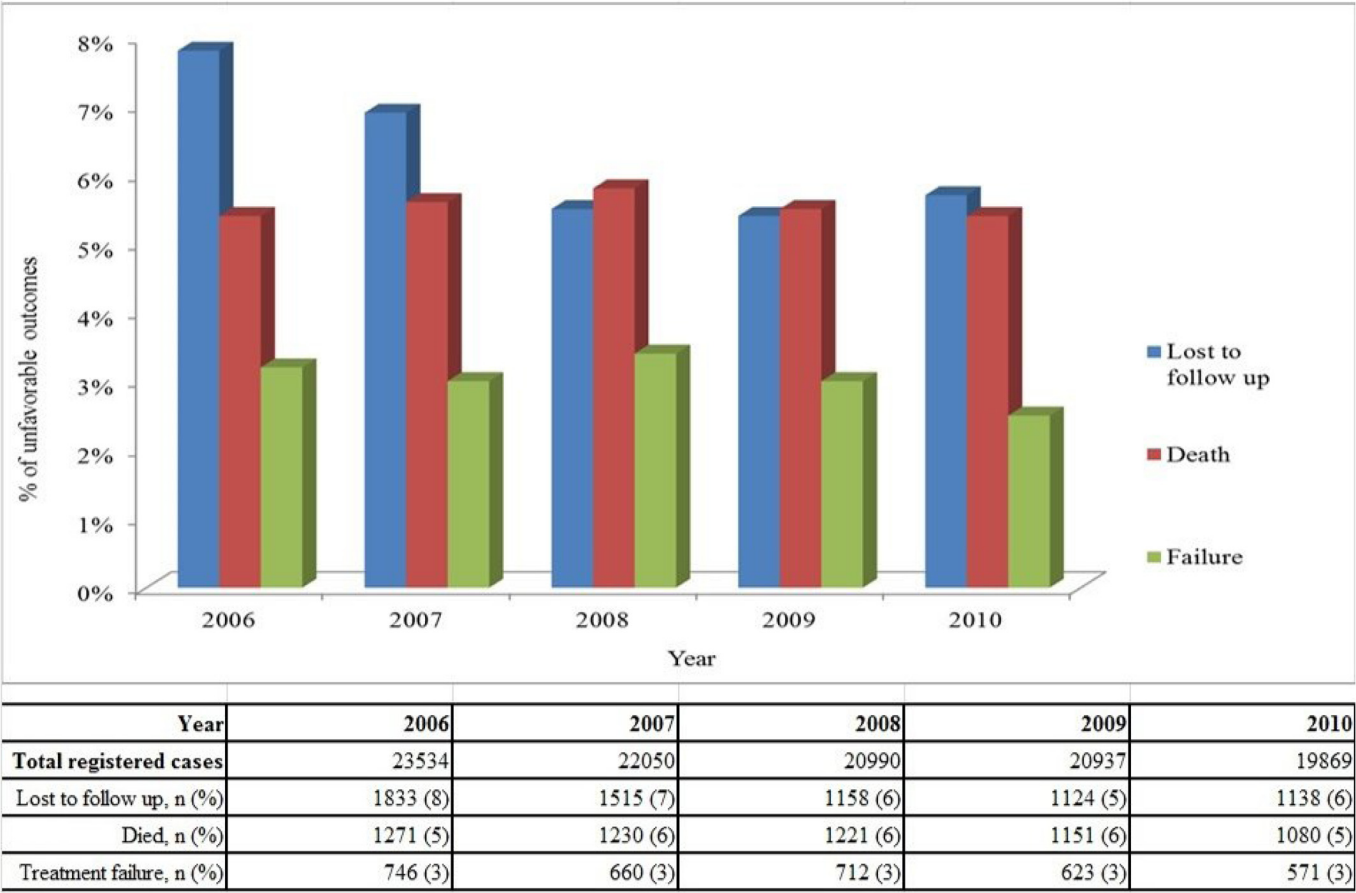
Unfavorable treatment outcomes over five years in Uzbekistan 2006–2010.

Overall, 83% (89622) of patients were successfully treated (cured or treatment completed), 6% (5953) died, 6% (6768) were lost to follow up, 3% (3312) failed treatment and 2% (1725) were transferred out. Trends in the different unfavorable outcomes between 2006 and 2010 are shown in Fig 1. Deaths and treatment failures have remained stable over time, while lost to follow up decreased from 7.8% in 2006 to 5.7% in 2010. Table 2 shows a breakdown of treatment outcomes by TB type and TB retreatment history. Of note, new smear positive cases had a treatment success rate of 82%, new EPTB cases had a treatment success rate of 93%, and retreatment cases had a treatment success rate of 73%.

**Table 2:** Treatment outcomes of all registered tuberculosis patients by TB type and treatment history, Uzbekistan 2006–2010.

	n	Treatment success n (%)	Diedn (%)	Treatment Failuren (%)	Lost to follow up n (%)	Transferred out n (%)
**New cases**						
sssssssssSmear positive	24480	19971 (82)	1437 (6)	1322 (5)	1257 (5)	493 (2)
Smear negative	32240	28198 (87)	1513 (5)	405 (1)	1750 (5)	374 (1)
No sputum/no result	1940	1572 (81)	129 (7)	100 (5)	101 (5)	38 (1)
Extrapulmonary	22356	20704 (93)	414 (2)	31 (0.1)	1054 (5)	153 (0.7)
**Retreatment cases**	26364	19177 (73)	2460 (9)	1454 (6)	2606 (10)	667 (3)
***Total***	107380	89622 (83)	5953 (6)	3312 (3)	6768 (6)	1725 (6)

### Factors associated with deaths, lost to follow-ups and treatment failures

Based on a multivariate analysis, factors found to be associated with death included being elderly (>55), being male, living in an urban area, having smear positive PTB, having a history of TB treatment, being HIV positive and being jobless, a pensioner or handicapped. The three provinces with the highest mortality were Samarkand province, Surkhandarya province and Tashkent city, (Table 3). Being a child or adolescent, and having EPTB, was protective for dying.

**Table 3:** Factors associated with deaths among TB patients in Uzbekistan 2006–2010.

*Variable*	n	Deaths n (%)	Crude OR (95% CI)	Adjusted OR^a^ (95% CI)	*P*-value
**Total**	107380	5953 (6)	-	-	-
**Age (years)**					
Children (<15)	11519	75 (1)	0.1 (0.09–0.1)	0.3 (0.2–0.5)	<0.001
Adolescent (15–18)	4764	96 (2)	0.4 (0.3–0.5)	0.7 (0.5–0.9)	0.005
Adults (19–55)	71522	3759 (5)	1	1	
Elderly patients (>55)	19575	2023 (10)	2.1 (2.0–2.2	1.9 (1.6–2.1)	<0.001
**Sex**					
Male	63724	3855 (6)	1.3 (1.2–1.3)	1.3 (1.2–1.4)	0.001
Female	43656	2098 (5)	1	1	
**Place of residence**					
Urban	32752	2257 (7)	1.5 (1.4–1.6)	1.3 (1.2–1.4)	<0.001
Rural	70705	3343 (5)	1	1	
Unknown	3923	353 (9)	2.0 (1.8–2.2)		
**Province**					
Rep. of Karakalpakstan	13905	702 (5)	1.7 (1.4–2.0)	1.1 (0.9–1.4)	0.40
Tashkent city	8504	849 (10)	3.5 (2.9–4.2)	1.9 (1.5–2.4)	0.001
Andijan province	8525	399 (5)	1.5 (1.3–1.9)	1.3 (1.0–1.6)	0.07
Bukhara province	4857	220 (4)	1.5 (1.2–1.9)	1.1 (0.9–1.5)	0.31
Jizzakh province	4873	147 (3)	1.0 (0.8–1.2)	0.9 (0.7–1.3)	0.69
Kashkadarya province	8593	279 (3)	1.1 (0.9–1.3)	1.2 (0.9–1.5)	0.17
Navoi province	4258	131 (3)	1	1	
Namangan province	8062	361 (4)	1.4 (1.2–1.8)	1.4 (1.1–1.8)	0.004
Samarkand province	11202	998 (9)	3.1 (2.6–3.7)	2.3 (1.8–2.9)	0.001
Surkhandarya province	5455	295 (5)	1.8 (1.5–2.2)	1.8 (1.4–2.3)	0.001
Syrdarya province	2601	144 (6)	1.8 (1.4–2.4)	1.7 (1.3–2.3)	0.001
Tashkent province	11314	622 (6)	1.8 (1.5–2.2)	1.6 (1.2–2.0)	0.001
Fergana province	9830	568 (6)	1.9 (1.6–2.3)	1.5 (1.2–1.9)	0.001
Khorezm province	5164	228 (4)	1.5 (1.2–1.8)	1.2 (0.9–1.5)	0.21
Navoi mining company	229	9 (4)	1.3 (0.6–2.6)	1.3 (0.6–2.7)	0.50
Unknown	8	1 (12)			
**HIV status**					
HIV positive	984	280 (29)	7.5 (6.5–8.7)	8.1 (6.9–9.5)	<0.001
HIV negative	96524	4852 (5)	1	1	
Unknown status	9872	821 (8.3)	1.7 (1.6–1.9)		
**TB type**					
PTB					
Smear positive	35178	2875 (8)	1.6 (1.5–1.7)	1.5 (1.5–1.7)	<0.001
Smear negative	44205	2277 (5)	1	1	
No sputum/no result	3303	293 (9)	1.8 (1.6–2.0)	1.6 (1.3–1.8)	<0.001
EPTB	24694	508 (2)	0.4 (0.4–0.4)	0.7 (0.6–0.8)	<0.001
**History of TB treatment**					
New cases	81016	3493 (4)	1	1	
Retreatment cases	26364	2460 (9)	2.3 (2.2–2.4)	1.7 (1.5–1.8)	<0.001
**Treatment category**					
0^b^	93	13 (14)	3.4 (1.9–6.2)		
I	76548	3454 (5)	1		
II	25986	2444 (10)	2.2 (2.1–2.3)		
III	4752	42 (1)	0.2 (0.1–0.3)		
Unknown	1	0 (0)			
**History of contact with TB patient**					
No	90673	4892 (5)	1.5 (1.3–1.7)		
Yes	5590	206 (4)	1		
Unknown	11117	855 (8)			
**History of imprisonment** ^**c**^					
No	44147	1893 (4)	1		
Yes	586	39 (7)	1.6 (1.1–2.2)		
Unknown	62647	4021 (6)	1.5 (1.4–1.6)		
**Occupational status**					
Worker	12105	381 (3)	1	1	
Jobless	48569	2476 (5)	1.7 (1.5–1.8)	1.5 (1.4–1.7)	<0.001
Pre-school age	2994	28 (1)	0.3 (0.2–0.4)	1.0 (0.5–1.9)	0.96
Pupil/student	11186	82 (1)	0.2 (0.2–0.3)	0.8 (0.6–1.1)	0.21
Pensioner	13898	1461 (11)	3.6 (3.2–4.1)	2.1 (1.8–2.5)	<0.001
Handicapped Unknown	5996 12632	585 (10) 940 (7)	3.3 (2.9–3.8) 2.5 (2.2–2.8)	2.4 (2.1–2.7)	<0.001

TB, Tuberculosis; PTB, Pulmonary TB; EPTB, Extrapulmonary TB; OR, Odds Ratio, CI, Confidence Interval

^a^Adjusted odds ratios only presented for variables included in the multivariate model; 92812 records included in the multivariate model

^b^0- patients who refused treatment, or treatment category not defined, or where TB diagnosis was based on the findings of a post-mortem

^c^Data should not be considered relevant as unknown cases more than 50% in this group of patients

Factors associated with lost to follow-up were being male, living in an urban area, being HIV positive, having previous TB treatment history, being jobless, and living in the following provinces: Bukhara province, Tashkent province, Andijan province, **(** Table 4 **)**. Being an adolescent was protective for being lost to follow-up.

**Table 4:** Factors associated with loss to follow-up among TB patients in Uzbekistan 2006–2010.

*Variable*	N	Loss to follow up n (%)	Crude OR (95% CI)	Adjusted OR^a^ (95% CI)	*P*-value
**Total**	107380	6768 (6)	-	-	-
**Age (years)**					
Children (<15)	11519	455 (4)	0.5 (0.5–0.6)	0.8 (0.6–1.0)	0.06
Adolescent (15–18)	4764	190 (4)	0.5 (0.5–0.6)	0.7 (0.6–0.8)	<0.001
Adults (19–55)	71522	5083 (7)	1	1	
Elderly patients (>55)	19575	1040 (5)	0.7 (0.7–0.8)	0.9 (0.8–1.0)	0.11
**Sex**					
Male	63724	4645 (7)	1.5 (1.5–1.6)	1.4 (1.4–1.5)	<0.001
Female	43656	2123 (5)	1	1	
**Place of residence**					
Urban	32752	2800 (9)	1.8 (1.7–1.9)	1.8 (1.7–2.0)	<0.001
Rural	70705	3560 (5)	1	1	
Unknown	3923	408 (10)	2.2 (2.0–2.4)		
**Province**					
Republic of Karakalpakstan	13905	693 (5)	1.4 (1.2–1.7)	0.9 (0.7–1.2)	0.54
Tashkent city	8504	528 (6)	1.8 (1.5–2.1)	0.8 (0.6–1.0)	0.03
Andijan province	8525	722 (8)	2.5 (2.1–2.9)	2.1 (1.6–2.6)	<0.001
Bukhara province	4857	564 (12)	3.5 (2.9–4.2)	3.3 (2.6–4.1)	<0.001
Jizzakh province	4873	177 (4)	1.0 (0.8–1.3)	1.0 (0.7–1.3)	0.83
Kashkadarya province	8593	239 (3)	0.8 (0.6–0.9)	0.7 (0.6–0.9)	0.02
Navoi province	4258	154 (4)	1	1	
Namangan province	8062	467 (6)	1.6 (1.4–2.0)	1.4 (1.1–1.7)	0.01
Samarkand province	11202	585 (5)	1.5 (1.2–1.8)	1.1 (1.1–1.7)	0.008
Surkhandarya province	5455	377 (7)	2.0 (1.6–2.4)	1.8 (1.4–2.3)	<0.001
Syrdarya province	2601	116 (5)	1.2 (1.0–1.6)	1.0 (0.8–1.4)	0.93
Tashkent province	11314	1195 (11)	3.1 (2.7–3.7)	2.4 (1.9–3.0)	<0.001
Fergana province	9830	636 (7)	1.8 (1.5–2.2)	1.6 (1.3–2.0)	<0.001
Khorezm province	5164	300 (6)	1.6 (1.3–2.0)	1.4 (1.1–1.8)	0.01
Navoi mining company	229	11 (5)	1.3 (0.7–2.5)	1.1 (0.5–2.1)	0.85
Unknown	8	2 (25)	8.9 (1.8–44.4)		
**HIV status**					
HIV positive	984	81 (8)	1.5 (1.2–1.8)		
HIV negative	96524	5607 (6)	1		
Unknown	9872	1080 (11)	2.0 (1.9–2.1)		
**TB type**					
PTB					
Smear positive	35178	2416 (7)	1.1 (1.0–1.1)		
Smear negative	44205	2893 (7)	1		
No sputum/no result	3303	277 (7)	1.1 (0.9–1.2)		
EPTB	24694	1232 (5)	0.7 (0.7–0.8)		
**History of TB treatment**					
New cases	81016	4162 (5)	1	1	
Retreatment cases	26364	2606 (10)	2.0 (1.9–2.1)	1.8 (1.7–1.9)	<0.001
**TB treatment category**					
0^b^	93	7 (8)	1.5 (0.7–3.3)		
I	76548	3911 (5)	1		
II	25986	2580 (10)	2.0 (1.9–2.2)		
III	4752	270 (6)	1.1 (1.0–1.3)		
Unknown	1	0 (0)	-		
**History of contact with TB patient**					
No	90673	309 (6)	1.4 (1.3–1.5)		
Yes	5590	5415 (6)	1		
Unknown	11117	1044 (9)			
**History of imprisonment** ^**c**^					
No	44147	2614 (6)	1		
Yes	586	62 (11)	1.9 (1.4–2.5)		
Unknown	62647	4092 (7)	1.1 (1.1–1.2)		
**Occupational status**					
Worker	12105	531 (4)	1	1	
Jobless	48569	3492 (7)	1.7 (1.5–1.9)	1.7 (1.6–1.9)	<0.001
Pre-school age	2994	140 (5)	1.1 (0.9–1.3)	1.5 (1.1–2.0)	0.02
Pupil/student	11186	399 (4)	0.8 (0.7–0.9)	1.2 (0.9–1.5)	0.25
Pensioner	13898	674 (5)	1.1 (1.0–1.2)	1.3 (1.1–1.6)	0.002
Handicapped	5996	423 (7)	1.7 (1.5–1.9)	1.3 (1.1–1.5)	0.001
Unknown	12632	1109 (9)	2.1 (1.9–2.3)		

TB, Tuberculosis; PTB, Pulmonary TB; EPTB, Extrapulmonary TB; OR, Odds Ratio, CI, Confidence Interval

^a^Adjusted odds ratios only presented for variables included in the multivariate model; 92812 records included in the multivariate model

^b^0- patients who refused treatment, or treatment category not defined, or where TB diagnosis was based on the findings of a post-mortem

^c^Data should not be considered relevant as unknown cases more than 50% in this group of patients

Factors associated with treatment failure included being adolescent, urban area population, having positive sputum result, previous treatment history, being HIV negative, being jobless, and residing in the following provinces: Fergana province, Tashkent city, Republic of Karakalpakstan, (Table 5). Being a child and having EPTB, was protective for failing treatment.

**Table 5:** Factors associated with treatment failure in TB patients in Uzbekistan, 2006–2010.

*Variables*	n (%)	Treatment failure n (%)	Crude OR (95% CI)	Adjusted OR^a^ (95% CI)	*P*-value
**Total**	107380	3312 (3)			
**Age (years)**					
Children (<15)	11519	32 (<1)	0.07 (0.05–0.1)	0.6 (0.3–0.9)	0.02
Adolescent (15–18)	4764	170 (4)	0.9 (0.8–1.1)	1.3 (1.1–1.7)	0.01
Adults (19–55)	71522	2716 (4)	1	1	
Elderly patients (>55)	19575	394 (2)	0.5 (0.5–0.6)	0.8 (0.7–1.0)	0.07
**Sex**					
Male	63724	2070 (3)	1.2 (1.1–1.2)		
Female	43656	1242 (3)	1		
**Place of residence**					
Urban	32752	1462 (5)	2.0 (1.9–2.2)	1.5 (1.4–1.7)	<0.001
Rural	70705	1590 (2)	1	1	
Unknown	3923	260 (7)	3.2 (2.7–3.5)		
**Province**					
Rep of Karakalpakstan	13905	682 (5)	3.6 (2.7–4.5)	2.6 (1.9–3.7)	<0.001
Tashkent city	8504	427 (5)	3.6 (2.7–4.7)	2.1 (1.4–2.9)	0.001
Andijan province	8525	304 (4)	2.5 (1.9–3.3)	1.9 (1.4–2.8)	<0.001
Bukhara province	4857	78 (2)	1.1 (0.8–1.5)	1.2 (0.8–1.8)	0.42
Jizzakh province	4873	61 (1)	0.9 (0.6–1.2)	0.6 (0.4–0.9)	0.02
Kashkadarya province	8593	17 (<1)	0.1 (0.1–0.2)	0.1 (0.1–0.2)	<0.001
Navoi province	4258	62 (2)	1	1	
Namangan province	8062	181 (2)	1.6 (1.2–2.1)	1.4 (1.0–2.0)	0.09
Samarkand province	11202	239 (2)	1.5 (1.1–2.0)	1.4 (1.0–1.9)	0.09
Surkhandarya province	5455	79 (2)	1.0 (0.7–1.4)	0.8 (0.5–1.1)	0.20
Syrdarya province	2601	85 (3)	2.3 (1.6–3.2)	1.7 (1.1–2.5)	0.01
Tashkent province	11314	492 (4)	3.1 (2.4–4.0)	2.1 (1.5–3.0)	<0.001
Fergana province	9830	447 (5)	3.2 (2.5–4.2)	2.8 (2.0–3.9)	<0.001
Khorezm province	5164	151 (3)	2 (1.5–2.7)	1.2 (0.8–1.8)	0.27
Navoi mining	229	6 (3)	1.8 (0.8–4.3)	2.7 (1.1–6.5)	0.03
Unknown	8	0 (0)	-	-	
**HIV status**					
Positive	984	23 (2)	1	1	
Negative	96524	2656 (3)	1.2 (0.8–1.8)	1.6 (1.1–2.5)	0.02
Unknown	9872	635 (7)	2.9 (1.9–4.4)		
**TB type**					
PTB					
Smear positive	35178	2482 (7)	5.5 (5.0–6.0)	5.5 (4.9–6.0)	<0.001
Smear negative	44205	606 (1)	1	1	
No sputum/no result	3303	192 (6)	4.4 (3.8–5.2)	4.3 (3.5–5.3)	<0.001
EPTB	24694	32 (0.1)	0.09 (0.07–0.1)	0.2 (0.1–0.2)	<0.001
**History of TB treatment**					
New cases	81016	1858 (2)	1	1	
Retreatment cases	26364	1454 (6)	2.5 (2.3–2.7)	1.7 (1.5–1.8)	<0.001
**Treatment category**					
0^b^	93	1 (1)	0.4 (0.06–3.1)		
I	76548	1856 (2)	1		
II	25986	1447 (6)	0.07 (0.03–0.1)		
III	4752	8 (0.2)	-		
Unknown	1	1 (0)			
**TB contact**					
No	90673	2425 (3)	1.6 (1.4–1.8)	1.6 (1.4–1.8)	<0.001
Yes	5590	887 (6)	1	1	
Unknown	11117	0 (0)			
**History of imprisonment** ^**c**^					
No	44147	1037 (2)	1		
Yes	586	19 (3)	1.4 (0.9–2.2)		
Unknown	62647	2256 (4)	1.6 (1.4–1.7)		
**Occupational status**					
Worker	12105	345 (3)	1	1	
Jobless	48569	1712 (4)	1.3 (1.1–1.4)	1.1 (1.0–1.3)	0.03
Pre-school age	2994	1 (0.03)	0.01 (0.002–0.8)	0.1 (0.01–0.8)	0.03
Pupil/student	11186	103 (0.9)	0.3 (0.3–0.4)	0.9 (0.6–1.2)	0.37
Pensioner	13898	225 (2)	0.6 (0.5–0.7)	0.7 (0.5–0.9)	0.005
Handicapped	5996	247 (4)	1.5 (1.2–1.7)	1.1 (0.9–1.3)	0.22
Unknown	12632	679 (6)	2 (1.7–2.3)	-	-

TB, Tuberculosis; PTB, Pulmonary TB; EPTB, Extrapulmonary TB; OR, Odds Ratio, CI, Confidence Interval

^a^Adjusted odds ratios only presented for variables included in the multivariate model; 92055 records included in the multivariate model

^b^0- patients who refused treatment, or treatment category not defined, or where TB diagnosis was based on findings of a post-mortem

^c^Data should not be considered relevant as unknown cases more than 50% in this group of patients

## Discussion

This is the first report from Uzbekistan, and one of the first from a former Soviet Union Country, describing the association of risk factors with treatment outcomes of the patients under first-line drug regimen over the course of five years. Systematic reviews have highlighted that there are limited large-scale data on TB treatment outcomes. Another systematic review of national-level TB treatment outcomes among the 30 European Union /European Economic Area countries indicated the same [9]. The WHO Global TB Report provide such data at national level; however detailed analysis of individual patient data, as well as associations between unfavorable treatment outcomes and selected demographic and clinical characteristics, are not reported [5].

Our study has shown promising treatment success (83%) among registered TB patients on first line treatment in Uzbekistan, with these data closely corroborating the data on treatment outcomes reported by WHO for Uzbekistan [5]. The overall treatment success rate that we report is marginally higher that reported by WHO because WHO only considers new smear positive cases in its analysis, whereas we have reported on all registered patients. This includes new smear negative and EPTB cases who have higher rates of treatment success than new smear positive cases. Moreover, we have been able to describe in detail patient characteristics together with certain factors that are associated with unfavorable treatment outcomes. These findings may help the national programme to define strategies and targeted interventions for the most vulnerable populations in order to further improve TB control.

Trends in unfavorable TB outcomes remained relatively stable over the five year period. Interestingly however, despite stable treatment failure rates, a recent DRS survey revealed alarmingly high rates of DR TB among new and previously treated cases [3]. Various factors may underpin this situation, including low case detection rate. Recent estimates presented in the latest WHO Global TB Report [5] suggest that only 50% of TB cases in Uzbekistan are being detected and put on ‘registered’ treatment. This indicates that a large number of TB cases are either going untreated or, due to factors such as stigma, are seeking treatment from ‘unofficial’ sources, the latter of which may be associated with patients receiving inefficacious treatment regimens. We can only speculate, but ‘unregistered’ cases may be a prime driver of the MDR-TB epidemic and this calls for urgent measures to improve case detection in Uzbekistan. In the context of Uzbekistan, this could be addressed by a) strengthening the capacity of primary health care facilities in TB case detection, given that these facilities are usually the first point of contact for most patients; b) improving the performance of microscopy laboratories located in rural places; c) raising community awareness on TB. During the study period, access to MDR-TB diagnosis and treatment was only guaranteed for patients residing in Tashkent city, Nukus city and in penitentiary system. It is encouraging to see that since 2013, MDR-TB treatment has become available for everyone in the country and, since 2014, Xpert MTB/RIF has become available in all provinces.

In our analysis we went beyond the usual convention of just stratifying patients according to whether they were adults or children, and also considered adolescents (15–18 years) and elderly patients (>55 years)–two groups that are rarely examined despite there being evidence that these are particularly vulnerable sub-groups in the context of other diseases [10]. In our study, adolescents had a higher likelihood of treatment failure and elderly patients had higher mortality. The latter has been reported in other settings [11,12,13] and may be due to age-related factors such as co-existing morbidities, like diabetes mellitus (which has been shown to increase the case fatality rate during TB treatment) [14], immunosuppression and a greater likelihood of unfavorable drug reactions [15,16].

The degree of unfavorable treatment outcomes between provinces varied quite notably, with higher mortality, lost to follow-up and treatment failure observed in the capital city Tashkent, Fergana, and in the Republic of Karakalpakstan which is also known to have the highest rate of drug-resistant TB in the world [3, 17, 18]. The differences between provinces may be explained by a number of factors such as i) variations in drug resistance prevalence and resistance patterns (for example, the highest rates of MDR-TB have been reported in the Republic of Karakalpakstan [3] which in turn, is the province with the highest treatment failure; in contrast, the lowest rates of MDR-TB are reported in Surkhandarya province which concordantly has a relatively low odds of treatment failure compared with other provinces, ii) the performance of the local programme in terms of directly observed treatment (DOT) management, (i.e. poor supervision at the primary health facilities of patients’ anti-TB drug intake during the continuation phase of treatment), iii) the performance of the local primary health care services in relation to TB case detection of TB through microscopy, iv) the availability of first and second line anti TB drugs on the open market (which may be inappropriately used by doctors (private and public based) who are treating ‘unregistered’ TB patients (NB. This is forbidden by national regulations) [6], and/or v) patient characteristics such as migration and population mobility. Operational research at the level of the province may help to identify these specific factors. Qualitative research methods in particular (such as direct observation, in-depth interviews with local key stake holders, and “content analysis” of local reports and archives), may provide additional information that quantitative data currently do not reveal. We have hypothesized several factors; of these, differences in performance between provinces, availability of TB-drugs on the “open market”, and migration and mobility patterns, could be explored with qualitative or mixed methods.

There were disparities across all unfavorable outcomes when comparing urban and rural areas. This finding has not been reported in other similar settings and thus we can only speculate on possible reasons for it. Deaths, lost to follow-ups, and treatment failures were all more common among urban than rural patients. Possible reasons for this may be related to differences in patients and/or differences in TB control activities between urban and rural areas. Co-morbidities such as diabetes mellitus may be more prevalent in urban rather than rural communities [19], the latter of which is known to be associated with a higher likelihood of unfavorable TB outcomes [20]. Identifying what these specific reasons are would require further investigation.

As shown in previous studies [21, 22, 23], treatment outcomes were worse among HIV-positive TB patients compared to HIV negative TB patients, especially death. In contrast, HIV-positive TB patients had lower lost to follow-up and treatment failure. This could be partially accounted for by the higher mortality among HIV positive TB patients–i.e. these patients are more likely to die before it becomes evident that they have been lost to follow-up or failed treatment [24]; it may also reflect better adherence to treatment among these patients. It is important to note that HIV-testing uptake was high in this national TB cohort and the country should maintain and further improve this, even though Uzbekistan is a low prevalence country.

Our study shows that treatment outcomes are poor among pulmonary sputum positive cases, and among previously treated patients rather than patients with negative sputum results and new TB cases. Our findings reflect what many other studies have shown. [25, 26]

The main strength of the study relates to the large countrywide size and national representativeness of the data. It is one of the first studies to have used countrywide TB data to assess risk factors associated with unfavorable treatment outcomes, and to have analyzed individual patient data rather than aggregate data. Most national TB programmes report only aggregate data as maintaining electronic databases that collect individual patient data is too resource demanding.

There are several study limitations. First, the study was reliant on routinely collected data which may have been subject to reporting errors typically encountered in programmatic settings (such as incomplete data, inaccurate data, typing errors etc.). Second, we were not able to analyze some particularly interesting subgroups of patients such as inmates, as large amounts of these data were incomplete. Finally, a major limitation was the misclassification of ‘transfer-outs’. Between 2003 and 2005, two MDR-TB pilot programmes were started in Karakalpakstan and the capital city Tashkent. Patients diagnosed with MDR-TB were transferred to the pilot clinics and their records were transferred to the MDR-TB register. In many instances, these patients were classified in the national database as “transferred out” rather than “treatment failure”. Furthermore, for patients who were transferred out to a different distict/province, the national database should have been updated to reflect the final outcome for that patient. This however never happened. Therefore, in this study the outcome “transferred out” consisted of patients for whom the final outcome was not ascertained and patients who were transferred into the MDR TB register after failing standard treatment. As such, a proportion of the transfer outs were essentially treatment failures, although this proportion remains unknown. To take account of this discrepancy we ran a sensitivity analysis in which all transfer outs were considered to be failures. When transfer-outs were combined with failures like this however, there were no notable differences in the factors associated with this combined outcome in comparison to those factors identified as being associated with treatment failure alone. This misclassification in standard treatment outcome reporting needs to be addressed going forwards.

In conclusion, this study has demonstrated how countrywide data can be used to monitor trends in TB outcomes and guide the NTP in identifying areas where targeted strategies need to be deployed for vulnerable groups and in certain parts of the country. We also highlight the need to unify the monitoring and reporting of TB outcomes between the national database for standard TB treatment and the database for MDR-TB. Finally, as migration between countries continues to increase surveillance of treatment failures and coordination of TB case management between neighboring countries needs to be reinforced.
